# Pattern Expression Nonnegative Matrix Factorization: Algorithm and Applications to Blind Source Separation

**DOI:** 10.1155/2008/168769

**Published:** 2008-06-12

**Authors:** Junying Zhang, Le Wei, Xuerong Feng, Zhen Ma, Yue Wang

**Affiliations:** ^1^School of Computer Science and Engineering, Xidian University, Xi'an 710071, China; ^2^Department of Mathematics and Computer Science, Valdosta State University, Valdosta, GA 31698, USA; ^3^The Bradley Department of Electrical and Computer Engineering, Virginia Polytechnic Institute and State University, VA 24061, USA

## Abstract

Independent component analysis (ICA) is a widely applicable and effective approach in blind source separation (BSS), with limitations that sources are statistically independent. However, more common situation is blind source separation for nonnegative linear model (NNLM) where the observations are nonnegative linear combinations of nonnegative sources, and the sources may be statistically dependent. We propose a pattern expression nonnegative matrix factorization (PE-NMF) approach from the view point of using basis vectors most effectively to express patterns. Two regularization or penalty terms are introduced to be added to the original loss function of a standard nonnegative matrix factorization (NMF) for effective expression of patterns with basis vectors in the PE-NMF. Learning algorithm is presented, and the convergence of the algorithm is proved theoretically. Three illustrative examples on blind source separation including heterogeneity correction for gene microarray data indicate that the sources can be successfully recovered with the proposed PE-NMF when the two parameters can be suitably chosen from prior knowledge of the problem.

## 1. Introduction

Blind source separation (BSS) is a very active topic recently in signal processing and neural network fields [1, 2]. It is an approach to recover the sources from their combinations (observations) without any understanding of how the sources are mixed. For a linear model, the observations are linear combinations of sources, that is, *X* = *A* *S*, where *S* is an *r* × *n* matrix indicating *r* source signals each in *n*-dimensional space, *X* is an *m* × *n* matrix showing *m* observations in *n*-dimensional space, and *A* is an *m* × *r* mixing matrix. Therefore, BSS problem is a matrix factorization, that is, to factorize observation matrix *V* into mixing matrix *A* and source matrix *S*.

Independent component analysis (ICA) has been found very effective in BSS for the cases where the sources are statistically independent. In fact, it factorizes the observation matrix *V* into mixing matrix *A* and source matrix *S* by searching the most nongaussianity directions in the scatter plot of observations, and has a very good estimation performance of the recovered sources when the sources are statistically independent. This is based on the Central Limit Theorem, that is, the distribution of a sum (observations) of independent random variables (sources) tends toward a Gaussian distribution under certain conditions. This induces the two serious constraints of ICA to the application of BSS: (1) the sources should be statistically independent to each other; (2) the sources should not follow Gaussian distribution. The performance of the recovered sources with ICA approach depends on the satisfactory of these two constraints, and decreases very rapidly when either of them is not satisfied. However in real world, there are many applications of blind source separation where the observations are nonnegative linear combinations of nonnegative sources, and the sources are statistically dependent to some extent. This is the model referred to as nonnegative linear model (NNLM), that is, *X* = *A* *S* with elements in both *A* and *S* nonnegative, and the rows in *S* (the sources) may be statistically dependent to some extent. One of the applications of this model is gene expression profiles, where each of the profiles, which is only in nonnegative values, represents a composite of more than one distinct but partially dependent sources [3], the profiles from normal tissue and from cancer tissue. What needs to be developed is an algorithm to recover dependent sources from the composite observations.

It is easy to recognize that BSS for NNLM is a nonnegative matrix factorization, that is, to factorize *X* into nonnegative *A* and nonnegative *S*, where nonnegative matrix factorization (NMF) technique is applicable. Several approaches have been developed on applying NMF-based technique for BSS of NNLM. For example, we proposed a method for decomposition of molecular signatures based on BSS of nonnegative dependent sources with direct usage of standard NMF [3]; Chichocki and his colleagues proposed a new algorithm for nonnegative matrix factorization in applications to blind source separation [4] by adding two suitable regularizations or penalty terms in the original objective function of the NMF to increase sparseness and/or smoothness of the estimated components. In addition, multilayer NMF was proposed by Cichocki and Zdunek for blind source separation [5], and nonsmooth nonnegative matrix factorization was proposed aiming at finding localized, part-based representations of nonnegative multivariate data items [6]. Some other researches include the work of Zdunek and Cichocki, who proposed to take advantage of the second-order terms of a cost function to overcome the disadvantages of gradient (multiplicative) algorithms for NMF for tackling the slow convergence problem of the standard NMF learning algorithms [7]; the work by Ivica Kopriva and his colleagues, who proposed a single-frame blind image deconvolution approach with nonnegative sparse matrix factorization for blind image deconvolution [8]; and the work by Liu and Zheng who proposed nonnegative matrix factorization-based methods for object recognition [9].

In this paper, we extend NMF to pattern expression NMF (PE-NMF) from the view point that the basis vector is desired to be the one which can express the data most efficiently. Its successful application to blind source separation of extended bar problem, nonnegative signal recovery problem, and heterogeneity correction problem for real gene microarray data indicates that it is of great potential in blind separation of dependent sources for NNLM model. The loss function for the PE-NMF proposed here is a special case of that proposed in [4], and here not only the learning algorithm for the proposed PE-NMF approach is provided, but also the convergence of the learning algorithm is proved by introducing some auxiliary function. For speeding up the learning procedure, a technique based on independent component analysis (ICA) is proposed, and has been verified to be effective for the learning algorithm to converge to desired solutions.

## 2. Pattern Expression NMF and BSS for NNLM Model

NMF problem is given a nonnegative *n* × *m* matrix *V*, find nonnegative *n* × *r* and *r* × *m* matrix factors *W* and *H* such that the difference measure between *V* and *W* *H* is the minimum according to some cost function, that is,

(1) V≈WH.

NMF is a method to obtain a representation of data using nonnegative constraints. These constraints lead to a part-based representation because they allow only additive, not subtractive, combinations of the original data. For the *i*th column of (1), that is, *v*_*i*_ = *W* *h*_*i*_, where *v*_*i*_ and *h*_*i*_ are the *i*th column of *V* and *H*, the *i*th datum (observation) is a nonnegative linear combination of the columns of *W* = (*W*_1_, *W*_2_,…, *W*_*r*_), while the combinatorial coefficients are the elements of *h*_*i*_. Therefore, the columns of *W*, that is, {*W*_1_, *W*_2_,…, *W*_*r*_}, can be viewed as the basis of the data *V* when *V* is optimally estimated by its factors.

### 2.1. Pattern Basis

**Figure 1:**
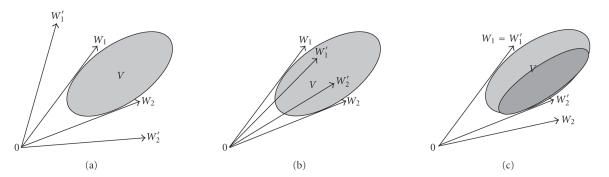
The basis {*W*_1_, *W*_2_}/{*W*_1_′, *W*_2_′} which obeys/violates (a) the first point; (b) the second point; (c) the third point in the definition of the pattern basis.

Let *W*_1_, *W*_2_,…, *W*_*r*_ be linearly independent *n*-dimensional vectors. We refer to the space spanned by arbitrarily nonnegatively linear combination of these *r* vectors the positive subspace spanned by *W*_1_, *W*_2_,…, *W*_*r*_. Then, *W*_1_, *W*_2_,…, *W*_*r*_ is the pattern expression of the data in this subspace, and is called the basis of the subspace. Evidently, the basis *W*_1_, *W*_2_,…, *W*_*r*_ derived from NMF is the pattern expression of the observation data in columns of *V*, but this expression may not be unique. Figure 1(a) shows an example of the data *V* which have two pattern expressions of {*W*_1_, *W*_2_} and {*W*_1_′, *W*_2_′}. Hence, we have the following questions: which basis is more effective in expressing the pattern of the observations in *V*? In order for the basis to express the pattern in *V* effectively, in our opinion, following three requirements should be satisfied:

1. the angle between the vectors in the basis should be as large as possible, such that each data in *V* is a nonnegatively linear combination of the vectors;
2. the angles between the vectors in the basis should be as small as possible to make the vectors clamp the data as tightly as possible, such that no space is left for expression of what is not included in *V*;
3. each vector in the basis should be of the most efficient in expression of the data in *V*, and the same efficient in this expression compared with any other vector in the basis.

The vectors defined with the above three requirements are what we call the pattern basis of the data, and the number of vectors in the basis, *r*, is called the pattern dimension of the data. Figures 1(a), 1(b), and 1(c) show, respectively, the too large between-angle, too small between-angles, and too unequally important basis situation with {*W*_1_′, *W*_2_′} as basis, where data in Figures 1(a) and 1(b) are assumed to be uniformly distributed in the gray area while those in Figure 1(c) are assumed to be nonuniformly distributed (the data in the dark gray area is denser compared with those in the light gray area). For these three cases, {*W*_1_, *W*_2_} is a better basis to express the data.

Notice that the second requirement in the definition of the pattern basis readily holds from the constraint of NMF that the elements in *H* are nonnegative. Then, we can get the three constraints as follows: (1) due to the requirement that the between-angle between each pair of vectors in the basis should be as large as possible, we have *W*_*i*_^*T*^ *W*_*j*_ → min, for *i* ≠ *j*, where *W_i_* is the *i*th column of the matrix *W*; (2) due to the requirement that each vector in the basis should be equally efficient in expression of the data in *V*, while the efficiency of the vector in this expression is measured by the summation of the projection coordinates of all the data in *V* to this vector, that is, samples *v*_*j*_, *j* = 1, 2,…, *n* if expressed in the vector *W*_*i*_, the efficiency of the vector *W*_*i*_ for expression of *v*_*j*_, *j* = 1, 2,…, *n* is ∑_*j* = 1_^*n*^ *h*_*ji*_, we have ∑_*i* = 1_^*r*^∑_*j* = 1_^*n*^ *h*_*ji*_ → min. Hence, we formulate PE-NMF problem as minimizing the loss function *E*(*W*, *H*; *α*, *β*) in the following equation subject to nonnegativity constraints.

**PE-NMF problem**

Given an *n* by *m* nonnegative observation matrix *V*, find an *n* by *r* and an *r* by *m* nonnegative matrix factors *W* and *H*, such that

(2) $$\begin{array}{ll} \underset{W,H}{\min}\,\;E(W,H;\alpha ,\beta ) & =\frac{1}{2}{∥V-WH∥}^{2}+\alpha \underset{i,j,\;j\;\neq \;i}{\sum }W_{i}^{T}W_{j}\\ & \;+\beta \underset{i,j}{\sum }h_{ij},\;\text{s}.\text{t}.\;\;W\geq 0,\;H\geq 0, \end{array}$$

where *W* ≥ 0, *H* ≥ 0 indicates that both *W* and *H* are nonnegative matrices, respectively, *W_i_* is *i*th column of matrix *W*, and *h_ij_* is the element in the *i*th row and *j*th column of the matrix *H*.

This problem is a special case of the constrained optimization problem proposed in [4]:

(3) $$\begin{array}{ll} \underset{W,H}{\min}\,\;E(W,H;\alpha ,\beta ) & =\frac{1}{2}{∥V-WH∥}^{2}+\alpha J_{W}(W)\\ & \;+\beta J_{H}(H),\;\text{s}.\text{t}.\;\;W\geq 0,\;H\geq 0. \end{array}$$

### 2.2. PE-NMF Algorithm and its Convergence

For the derivation of learning algorithm for *W* and *H*, we first present and prove the following lemma.

**Lemma 1.**

*For any r by r symmetric nonnegative matrix* **Q** *and for any r-dimensional nonnegative row vector* **w**, *the r by r matrix*

(4) $$F=\delta_{ab}\frac{{\left(Qw^{T}\right)}_{a}}{w_{a}}-Q$$

*is always semipositive definite, where *δ*_*ab*_((*Qw*^*T*^)_*a*_/*w*_*a*_) represents a diagonal matrix with diagonal element in the ath row and ath column being (*Qw*^*T*^)_*a*_/*w*_*a*_.*

**Proof.**

By noticing that *w_a_* and *w_b_* are nonnegative, the definition of the matrix *F* is the same as that of the matrix *S* in which the *a*th row and *b*th column element is *S*_*ab*_ = *w*_*a*_ *F* *w*_*b*_. Hence, we consider proving the semipositive definition of the matrix *S* in the following context.

For any *r*-dimensional vector **V**, we have the following formula:

(5) $$\begin{array}{ll} V^{T}SV & =\underset{ab}{\sum }V_{a}S_{ab}V_{b}=\underset{ab}{\sum }V_{a}w_{a}Fw_{b}V_{b}\\ & =\underset{ab}{\sum }\left[V_{a}w_{a}\delta_{ab}\frac{(Qw^{T}{)}_{a}}{w_{a}}w_{b}V_{b}-V_{a}w_{a}Q_{ab}w_{b}V_{b}\right]\\ & =\underset{ab}{\sum }[V_{a}w_{a}\delta_{ab}\frac{(Qw^{T}{)}_{a}}{w_{a}}w_{b}V_{b}]-\underset{ab}{\sum }V_{a}w_{a}Q_{ab}w_{b}V_{b}\\ & =A-B, \end{array}$$

where *A* denotes the first term and *B* denotes the second term in the above formula. By noticing that *Q* is a symmetric matrix, we have (*Q* *w* ^*T*^)_*a*_ = (*w* *Q*)_*a*_, and hence the first term *A* becomes

(6) $$\begin{array}{ll} A & =\underset{a}{\sum }V_{a}w_{a}\frac{{\left(wQ\right)}_{a}}{w_{a}}w_{a}V_{a}\;=\underset{a}{\sum }{\left(wQ\right)}_{a}w_{a}V_{a}^{2}\\ & =\underset{a}{\sum }\left(\underset{b}{\sum }w_{b}Q_{ba}\right)w_{a}V_{a}^{2}=\underset{b}{\sum }\left(\underset{a}{\sum }w_{a}Q_{ab}\right)w_{b}V_{a}^{2}\\ & =\underset{ab}{\sum }w_{a}Q_{ab}w_{b}V_{a}^{2}, \end{array}$$

we substitute the above *A* into formula (5), and obtain

(7) $$\begin{array}{ll} V^{T}SV & =\underset{ab}{\sum }\left(w_{a}Q_{ab}w_{b}V_{a}-w_{a}Q_{ab}w_{b}V_{a}V_{b}\right)\\ & =\underset{ab}{\sum }w_{a}Q_{ab}w_{b}[V_{a}^{2}-V_{a}V_{b}]\\ & =\underset{ab}{\sum }w_{a}Q_{ab}w_{b}[\frac{1}{2}V_{a}^{2}+\frac{1}{2}V_{b}^{2}-V_{a}V_{b}]\\ & =\frac{1}{2}\underset{ab}{\sum }w_{a}Q_{ab}w_{b}{\left(V_{a}-V_{b}\right)}^{2}. \end{array}$$

Due to the fact that *w* and *Q* are a row vector and a nonnegative matrix, respectively, hence for any *r*-dimensional row vector *V*, we have

(8) $$V^{T}SV=\frac{1}{2}\underset{ab}{\sum }w_{a}Q_{ab}w_{b}{\left(V_{a}-V_{b}\right)}^{2}\geq 0.$$

Hence, the matrix *S* and therefore *F* = *δ*_*ab*_((*Q* *w* ^*T*^)_*a*_/*w*_*a*_) − *Q* is a semipositive definite matrix.

Now in Theorem 1, we derive learning algorithm and prove its convergence for updating each row *w* in *W* when *H* is set to be a fixed nonnegative matrix. The learning algorithm for updating each column *h* in *H* when *W* is set to be a fixed nonnegative matrix is depicted in Theorem 2, and can be proved similarly but skipped due to the limitation of the space.

**Theorem 1.**

*For the quadratic optimization problem,*

(9) $$\begin{array}{c} \underset{w}{\min}\,\;E(w;H,\alpha )=\frac{1}{2}{∥v-wH∥}^{2}+\frac{1}{2}\alpha wMw^{T},\;\text{s}.\text{t}.\;w\geq 0, \end{array}$$

*where *w* is an *r*-dimensional row vector, *v* is a given *m*-dimensional nonnegative row vector, *H* is an *r* by *m* fixed nonnegative matrix, *M* is an *r* by *r* constant matrix with all elements being 1 except diagonal elements being zeros, and *α* is a fixed nonnegative parameter. The following update algorithm*

(10) $$w_{a}^{t+1}=w_{a}^{t}\frac{{\left(vH^{T}\right)}_{a}}{{\left(w^{t}HH^{T}+\alpha w^{t}M\right)}_{a}}$$

*converges to its optimal solution from any initialized nonnegative vector *w*^0^.*

**Proof.**

The convergence proof will be performed by introducing an appropriate auxiliary function *F*(*w*, *w* ^*t*^) that satisfies

(11) $$F(w^{t},w^{t})=E\left(w^{t}\right),$$

(12) $$F(w,w^{t})\geq E(w).$$

If such a function can be found, then the update of *w* by setting

(13) $$w^{t+1}=\underset{w}{\arg\;\min}\,\;F(w,w^{t})$$

will make

(14) $$E(w^{t+1})\leq F(w^{t+1},w^{t})\leq F(w^{t},w^{t})=E(w^{t})$$

which always makes the objective function *E*(*w*) to be decreased with respect to iterations in the algorithm, indicating that the algorithm converges with the updating formula (13).

Now we construct the auxiliary function to be

(15) $$\begin{array}{ll} F(w,w^{t}) & =E(w^{t})+(w-w^{t})\nabla E(w^{t})\\ & \;+\frac{1}{2}(w-w^{t})J(w^{t}){\left(w-w^{t}\right)}^{T}, \end{array}$$

where *J*(*w* ^*t*^) is a diagonal matrix

(16) $$J(w^{t})=\delta_{ab}\frac{{\left(HH^{T}w^{tT}+\alpha Mw^{tT}\right)}_{a}}{{\left(w^{tT}\right)}_{a}}.$$

Obviously, *F*(*w* ^*t*^, *w* ^*t*^) = *E*(*w* ^*t*^), so formula (11) holds.

The Taylor expansion of the loss function *E*(*w*), when *w* approaches *w* ^*t*^, can be written to be

(17) $$\begin{array}{ll} E(w) & =E(w^{t})+(w-w^{t})\nabla E(w^{t})\\ & \;+\frac{1}{2}(w-w^{t})(HH^{T}+\alpha M){\left(w-w^{t}\right)}^{T}. \end{array}$$

By subtracting *F*(*w*, *w* ^*t*^) in (8)–(16) to *E*(*w*) in (7)–(17), we have

(18) $$\begin{array}{ll} F(w,w^{t})-E(w) & =\frac{1}{2}(w-w^{t})[\delta_{ab}\frac{{\left(HH^{T}w^{tT}+\alpha Mw^{tT}\right)}_{a}}{{\left(w^{tT}\right)}_{a}}-HH^{T}-\alpha M]{\left(w-w^{t}\right)}^{T}\\ & =\frac{1}{2}(w-w^{t})[\delta_{ab}\frac{{\left(Qw^{tT}\right)}_{a}}{{\left(w^{tT}\right)}_{a}}-Q]{\left(w-w^{t}\right)}^{T}, \end{array}$$

where *Q* = *H* *H* ^*T*^+*α* *M*. Due to the fact that *Q* is a nonnegative symmetric matrix since *H* is the nonnegative factor of *V*, and *α* is always a nonnegative parameter, and the fact that *w* ^*t*^ is a nonnegative vector, we have, from Lemma 1, that the matrix *δ*_*ab*_((*Q* *w* ^*tT*^)_*a*_/(*w* ^*tT*^)_*a*_) − *Q* is semipositive definite, and therefore we always have *F*(*w*,*w* ^*t*^) − *E*(*w*) ≥ 0. Hence, updating *w* according to *w* ^*t*+1^ = arg min_*w*_ *F*(*w*, *w* ^*t*^) always leads the iteration process to converge.

We employ the steepest descent search strategy for optimal *w*. For this purpose, we have *w* ^*t*+1^ to satisfy (∂*F*(*w*, *w* ^*t*^))/∂*w*|_*w* = *w*^*t* + 1^_ = 0, from which we get ∇*E*(*w* ^*t*^) + *J*(*w* ^*t*^)(*w* ^*t*+1^ − *w* ^*t*^)^*T*^ = 0, or equally

(19) $$w^{t+1T}=w^{tT}-J^{-1}(w^{t})\nabla E(w^{t}).$$

By the definition of the loss function *E*(*w*), we have

(20) $$\begin{array}{ll} \nabla E(w^{t}) & =H(H^{T}w^{t}-v^{T})+\alpha Mw^{tT}\\ & =(HH^{T}+\alpha M)w^{tT}-Hv^{T}. \end{array}$$

Since *J*(*w* ^*t*^) is a diagonal matrix, we only need to compute inversion of each diagonal element in J for *J* ^−1^. Hence, we have the following updating formula for the *a*th element of *w*:

(21) $$\begin{array}{ll} w_{a}^{t+1} & =w_{a}^{t}-\frac{{\left(w^{tT}\right)}_{a}}{{\left(HH^{T}w^{tT}+\alpha Mw^{tT}\right)}_{a}}\\ & \;\cdot {\left(HH^{T}w^{tT}+\alpha Mw^{tT}-Hv^{T}\right)}_{a}\\ & =w_{a}^{t}\frac{{\left(Hv^{T}\right)}_{a}}{{\left(HH^{T}w^{tT}+\alpha Mw^{tT}\right)}_{a}}\\ & =w_{a}^{t}\frac{{\left(vH^{T}\right)}_{a}}{{\left(w^{t}HH^{T}+\alpha w^{t}M\right)}_{a}}. \end{array}$$

**Theorem 2.**

*For the quadratic optimization problem.*

(22) $$\begin{array}{c} \underset{h}{\min}\,\;E(h;W,\beta )=\frac{1}{2}∥v-Wh{∥}^{2}+\beta I^{T}h,\;\text{s}.\text{t}.\;h\geq \text{0,} \end{array}$$

*where h is r-dimensional column vector, v is a given n-dimensional nonnegative column vector, W is an n by r fixed nonnegative matrix, I is an *r* × *m* matrix with all the elements being 1s, and *β* is a fixed nonnegative parameter. The following rule*

(23) $$h_{a}^{t+1}=h_{a}^{t}\frac{{\left(W^{T}v\right)}_{a}}{{\left(W^{T}Wh^{t}+\beta I\right)}_{a}}$$

*converges to its optimal solution from any initialized nonnegative vector *h*^0^.*

This theorem can be proved similarly as the proof of Theorem 1.

By representing (10) and (23) in (elementwise) Hadamard product, one has the following learning algorithm for updating both W and H for the PE-NMF optimization problem in (1).

**Theorem 3.**

*For the optimization problem shown in (1), the above learning algorithm converges to locally optimal solution from any initialized nonnegative vector *H*^0^ and *W*^0^.*

It is evident that the portion relating to the row w in the objective function *E*(*W*, *H*; *α*, *β*) in (1) is just *E*(*w*; *H*, *α*) in (9), and the portion relating to the column h in the objective function *E*(*W*, *H*; *α*, *β*) in (1) is just *E*(*h*; *W*, *β*) in (22). Hence, using formula to update w and h alternatively will make the learning process to converge to the solution of the objective function *E*(*W*, *H*; *α*, *β*). Hence, the above theorem can be easily proved on the basis of Theorems 1 and 2. 

The update of the *W* and *H* can also be expressed with MatLab command of *W* = *W*. ∗ (*V* ∗ *H*′)./(*W* ∗ *H* ∗ *H*′ + alfa ∗ *W* ∗ *M*) and *H* = *H*. ∗ (*W*′ ∗ *V*)./(*W* ′ ∗ *W* ∗ *H* + beta).

### 2.3. Initialization of the Algorithm

To our knowledge, it seems that there are two main reasons for NMF to converge to undesired solutions. One is that the basis of a space may not be unique theoretically, and therefore separate runs of NMF may lead to different results. Another reason may come from the algorithm itself, that the loss function sometimes gets stock into local minimum during its iteration. By revisiting the loss function of the proposed PE-NMF, it is seen that similar to NMF, the above PE-NMF still sometimes gets stock into local minimum during its iteration, and/or the number of iterations required for obtaining desired solutions is very large. For the sake of these, an ICA-based technique was proposed for initializing source matrix instead of setting it to be a nonnegative matrix at random: we performed ICA on the observation signals, and set the absolute of the independent components obtained from ICA to be the initialization of the source matrix. In fact, there are reasons that the resultant independent components obtained from ICA are generally not the original sources. One reason is the nonnegativity of the original sources but centering preprocess of the ICA makes each independent component be both positive and negative in its elements: the means of each independent component is zero. Another reason is possibly dependent or partially independent original sources which does not follow the independence requirement of sources in the ICA study. Hence, the resultant independent components from ICA could not be considered as the recovery of the original sources. Even so, they still provide clues of the original sources: they can be considered as *very rough* estimations of the original sources. From this perspective, and by noticing that the initialization of the source matrix should be nonnegative, we set the absolute of the independent components obtained from ICA as the initialization of the source matrix for the proposed PE-NMF algorithm. Our experiments indicate that such an initialization technique is very effective in speeding up the learning process for getting desired solutions.

## 3. Experiments and Results

The proposed PE-NMF algorithms have been extensively tested for many difficult benchmarks for signals and images with various statistical distributions. Three examples will be given in the following context for demonstrating the effectiveness of the proposed method compared with standard NMF method and/or ICA method. In ICA approach here, we decenteralize the recovered signals/images/microarrays for its nonnegativity property for compensating the centering preprocessing of the ICA approach. The NMF algorithm is simply the one proposed in [10] and the ICA algorithm is simply the FastICA algorithm generally used in many applications in [11]. The examples include blind source separation of extended bar problem, mixed signals, and real microarray gene expression data in which heterogeneity effect occurs.

### 3.1. Extended Bar Broblem

**Figure 2:**
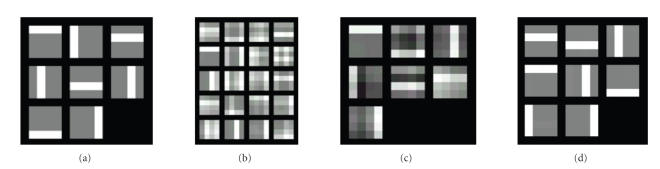
Bar problem solution obtained from NMF: (a) source images, (b) mixed images, (c) recovered images from ICA, and (d) recovered images from NMF.

**Figure 3:**
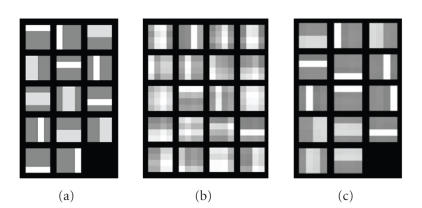
Extended bar problem solution obtained from PE-NMF: (a) source images, (b) mixed images, (c) recovered images from PE-NMF.

**Figure 4:**
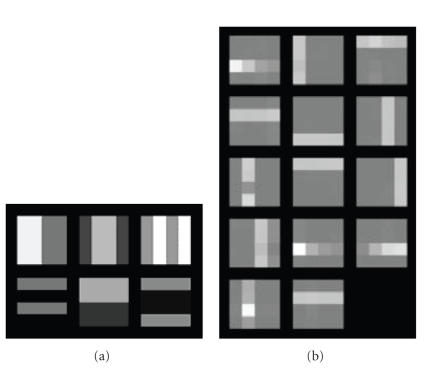
Recovered images from (a) ICA, and (b) NMF for the extended bar problem.

The linear bar problem [12] is a blind separation of bars from their combinations. 8 nonnegative feature images (sources) sized 4 × 4 including 4 vertical and 4 horizontal thin bar images, shown in Figure 2(a), are randomly mixtured to form 1000 observation images, the first 20 shown in Figure 2(b). The solution obtained from ICA and NMF with *r* = 8 are shown in Figures 2(c) and 2(d), respectively, indicating that NMF can fulfill the task very well compared with ICA . However, when we extended this bar problem into the one which is composed of two types of bars, thin one and thick one, NMF failed to estimate the original sources. For example, fourteen source images sized 4 × 4 with four thin vertical bars, four thin horizontal bars, three wide vertical bars, and three wide horizontal bars, shown in Figure 3(a), are nonnegative and evidently statistically dependent. These source images were randomly mixed with mixing matrix of elements arbitrarily chosen in [0, 1] to form 1000 mixed images, the first 20 shown in Figure 2(b). The PE-NMF with parameter *α* = 4 and *β* = 1 was performed on these mixed images for *r* = 14. The resultant images, which are shown in Figure 2(c), indicate that the sources were recovered successfully with the proposed PE-NMF. For comparison, many times we tried using ICA and NMF on this problem for avoiding obtaining local minimum solutions, but always failed to recover the original sources. Shown in Figures 4(a) and 4(b) are the examples of the recovered images with these two approaches. Notice that both the ones recovered from ICA and NMF are very far from the original sources, and even the number of sources estimated from the ICA is only 6, rather than 14. It is noticeable that the recovered images from the PE-NMF with some other parameter such as *α* = 4.2 and *β* = 0.1 are comparable to the ones shown in Figure 3(c), indicating that the proposed method is not very sensitive to the parameter selection for this example.

### 3.2. Recovery of Mixed Signals

**Figure 5:**
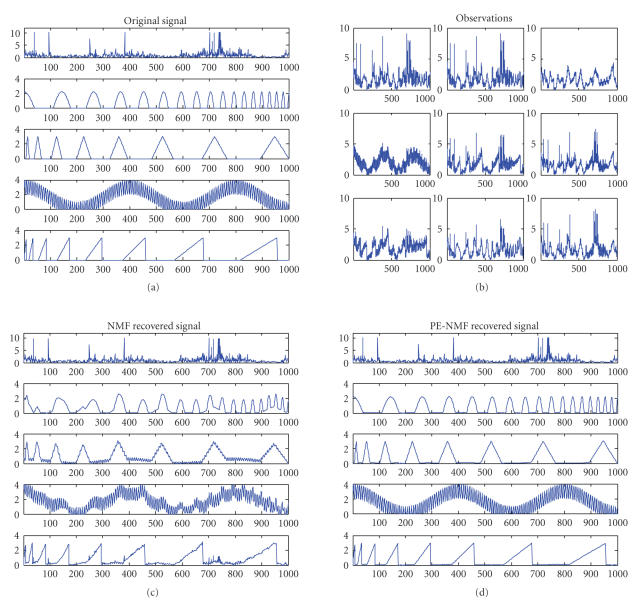
Blind signal separation example: (a) 5 original signals, (b) 9 observations, (c) recovered signals from NMF, and (d) recovered signals from PE-NMF.

We performed experiments on recovering 5 nonnegative signals from 9 mixtures of 5 nonnegative dependent source signals, which is the one in [4]. The 9 mixture observation signals come from arbitrarily nonnegative linear combinations of the 5 nonnegative source signals shown in Figure 5(a). The difficulty to recover the sources is a very small number of observations compared with the number of sources. Both NMF and our proposed PE-NMF (where *α* and *β* are taken to be 0.001 and 17.6, resp.) were employed for recovery of the sources. By comparison of the resultant signals obtained by NMF shown in Figure 5(c) and these obtained by PE-NMF shown in Figure 5(d), it is evident that the PE-NMF can recover the sources with a higher recovery performance. In fact, the signal-to-interference ratios (SIRs) for the recovered sources from NMF is only 22.17, 11.13, 10.98, 14.91, and 14.15 while that from PE-NMF increases to 47.10, 28.89, 26.67, 83.44, and 28.75 for the 5 source signals.

### 3.3. Heterogeneity Correction of Gene Micrroarrys

Gene expression microarrays promise powerful new tools for the large-scale analysis of gene expression. Using this technology, the relative mRNA expression levels derived from tissue samples can be assayed for thousands of genes simultaneously. Such global views are likely to reveal previously unrecognized patterns of gene regulation and generate new hypotheses warranting further study (e.g., new diagnostic or therapeutic biomarkers). However, as a common feature in microarray profiling, gene expression profiles represent a composite of more than one distinct but partially dependent sources (i.e., the observed signal intensity will consist of the weighted sum of activities of the various sources). More specifically, in the case of solid tumors, the related issue is called partial volume effect (PVE), that is, the heterogeneity within the tumor samples caused by stromal contamination. Blind application of microarray profiling could result in extracting signatures reflecting the proportion of stromal contamination in the sample, rather than underlying tumor biology. Such “artifacts” would be real, reproducible, and potentially misleading, but would not be of biological or clinical interest, while can severely decrease the sensitivity and specificity for the measurement of molecular signatures associated with different disease processes. Despite their critical importance to almost all the followup analysis steps, this issue, called partial volume correction (PVC), is often less emphasized or at least has not been rigorously addressed as compared to the overwhelming interest and effort in pheno/gene-clustering and class prediction.

**Figure 6:**
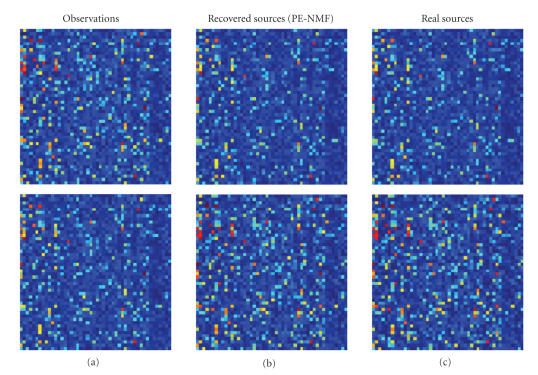
Heterogeneity correction result: (a) observations, (b) recovered sources from PE-NMF, and (c) real sources.

**Figure 7:**
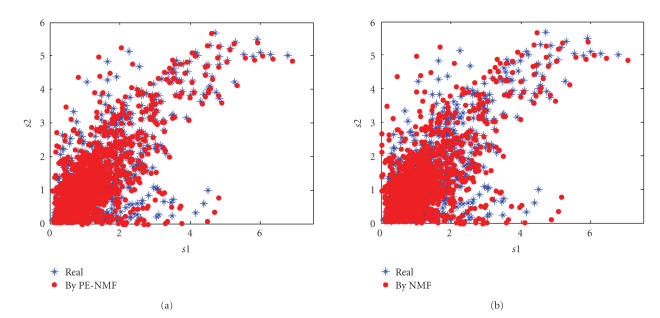
The scatter plots of the real sources (blue stars) and the recovered sources (red dots) from (a) PE-NMF, and (b) NMF.

The effectiveness of the proposed PE-NMF method was tested with real-world data set, microarray gene expression data set, for PVC. The data set consists of 2308 effective gene expressions from two samples of neuroblastoma and non-Hodgkin lymphoma cell tumors [13]. Two observation microarrays, recovered microarrays from PE-NMF, and two pure source microarrays are shown in Figures 6(a), 6(b), and 6(c), respectively. Notice that the true sources are determined, in our present case, by separately profiling the pure cell lines that provide the ground truth of the gene expression profiles from each cell populations. In our clinical case, we use laser-capture microdissection (LCM) technique to separate cell populations from real biopsy samples. By comparison of Figures 6(b) and 6(c), the blind source separation by PE-NMF method recovered the pure microarray successfully. Figures 7(a) and 7(b) show the scatter plots of the recovered microarrays from PE-NMF and from NMF compared with these of the pure microarrays. These scatter plots and the SIRs of being 56.79 and 31.73 for the PE-NMF approach and of being only 21.20 and 32.81 for the NMF approach also indicate that the proposed PE-NMF is effective in recovering the sources successfully. Many other independent trials using other gene sets reached a similar result.

## 4. Conclusions

This paper proposes a pattern expression nonnegative matrix factorization (PE-NMF) approach for efficient pattern expression and applies it to blind source separation for nonnegative linear model (NNLM). Its successful application to blind source separation of extended bar problem, nonnegative signal recovery problem, and heterogeneity correction problem for real microarray gene data indicates that it is of great potential in blind source separation problem for NNLM model. The loss function for the PE-NMF proposed here is in fact an extension of the multiplicative update algorithm proposed in [10], with the two terms introduced with parameters *α* and *β*, respectively, in which *β* is for update rule for the matrix *H*, which is similar to some sparse NMF algorithms [14], and *α* as the regularization term added to *H* *H* ^*T*^ in the update rule for matrix *W*. The loss function for the PE-NMF is a special case of that proposed in [4]. However, in this approach, not only the learning algorithm is motivated by expressing patterns more effectively and more efficiently, and experimented successfully in a wide range of applications, but also the convergence of the learning algorithm is proved by introducing some auxiliary function. In addition, a technique based on independent component analysis (ICA) is proposed for speeding up the learning procedure, and has been verified to be effective for the learning algorithm to converge to desired solutions.

Same as what has been mentioned in [4], the optimal choice of PE-NMF parameters depends on the distribution of data and a priori knowledge about the hidden (latent) components. However, our experimental results on extended bard problem indicate that the parameter choice is not so sensitive to some problems.

## Figures and Tables

**Algorithm 1:**
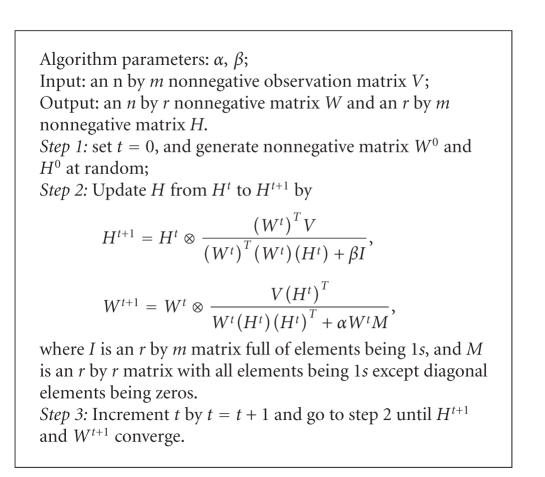
Learning algorithm.
